# Épidémiologie hospitalière des troubles psychiatriques au Mali

**DOI:** 10.11604/pamj.2022.41.160.30663

**Published:** 2022-02-23

**Authors:** Souleymane Papa Coulibaly, Housseini Dolo, Cyrielle Alexandra Malah Notue, Modibo Sangaré, Pakuy Pierre Mounkoro, Alhousseini Aboubacar, Joseph Traore, Aperou Eloi Dara, Kadiatou Traore, Mahamadou Kone, Boubacar Maiga, Souleymane Coulibaly, Youssoufa Maiga

**Affiliations:** 1 Faculté de Médecine et d´Odontostomatologie de Bamako, Bamako, Mali,; 2 Département de Psychiatrie, Centre Hospitalier Universitaire Point G, Bamako, Mali,; 3 Université des Sciences, des Techniques et des Technologies de Bamako, Bamako, Mali,; 4 Département de Neurologie, Centre Hospitalier Universitaire Gabriel Touré, Bamako, Mali

**Keywords:** Trouble psychiatrique, substance toxique, schizophrénie, Point G, Mali, Psychiatric disorder, toxic substance, schizophrenia, Point G, Mali

## Abstract

**Introduction:**

au Mali, il n'existe pas de données sur la prévalence des troubles mentaux. L´objectif de ce travail était de décrire les aspects cliniques et épidémiologiques des personnes hospitalisées en psychiatrie.

**Méthodes:**

notre étude était de type transversal conduite dans le service de psychiatrie du Centre Hospitalier Universitaire Point G sur 1105 dossiers de personnes hospitalisées pour trouble psychiatrique entre janvier 2014 et décembre 2018.

**Résultats:**

l'âge moyen était de 32,6± 11,1 ans avec des extrêmes de 13 et 82 ans. Le sexe masculin a représenté 83,8% (926/1105), 53,2% (588/1105) étaient célibataires, 18,8% (208/1105) n´avaient pas d´emploi et 28,2% (310/1105) avaient un niveau d'éducation primaire. Des antécédents de troubles psychiatriques étaient présents chez 74,0% (818/1105), la notion de consanguinité entre les parents était présente chez 22,7% (251/1105). L´utilisation de drogues était présente dans 42,9% (474/1105) dont le tabac avec 32,6% (361/1105), le cannabis avec 26,0% (287/1105) et/ou l'alcool avec 15,6% (172/1105). La demande de soins provenait des familles chez 87,5% (967/1105). L'agressivité était le motif de consultation le plus fréquent avec 44,5% (492/1105). Dans 67,8% (749/1105) des cas, le diagnostic était la schizophrénie, des troubles schizotypiques ou des troubles délirants. Les premiers recours aux soins étaient traditionnels avec 58,7% (649/1105).

**Conclusion:**

les personnes hospitalisées pour trouble psychiatrique de 2014 à 2018 étaient majoritairement jeunes et de sexe masculin avec un antécédent psychiatrique. Elles souffraient principalement de schizophrénie, de troubles schizotypiques et de troubles délirants.

## Abstract (en)

**Introduction:**

in Mali, there are no data on the prevalence of mental disorders. The purpose of this study was to describe the clinical and epidemiological features of patients hospitalised in the Department of Psychiatry.

**Methods:**

we conducted a cross-sectional study in the Department of Psychiatry at the University Hospital of Point G between January 2014 and December 2018. Data were collected from the medical records of 1105 patients hospitalised for psychiatric disorders.

**Results:**

the average age of patients was 32.6 ± 11.1 years, ranging from 13 to 82 years. Male patients accounted for 83.8% (926/1105) of enrolled subjects, 53.2% (588/1105) were single, 18.8% (208/1105) were not employed and 28.2% (310/1105) had primary education. Seventy-four percent (818/1105) had a history of psychiatric disorders, 22.7% (251/1105) were born to consanguineous parents. Drug use was reported in 42.9% (474/1105) of cases, including tobacco 32.6% (361/1105), cannabis 26.0% (287/1105) and/or alcohol 15.6% (172/1105). The demand for care came from families in 87.5% (967/1105) of cases. Aggression was the most common reason for consultation (44.5%; 492/1105). In 67.8% (749/1105) of cases, the diagnosis was schizophrenia, schizotypic disorders or delusional disorders. The first use of care was traditional in 58.7% (649/1105) of cases.

**Conclusion:**

people hospitalised for psychiatric disorders from 2014 to 2018 were predominantly young and male with a history of psychiatric disorders. They mainly had schizophrenia, schizotypic disorders and delusional disorders.

## Introduction

L´hospitalisation en psychiatrie est un moyen de soins très souvent assimilé aux urgences psychiatriques [1, 2]. Les patients admis en hospitalisation sont de divers profils [3]. Les crises d´agitations, d´agressivités, suicidaires et les symptômes psychotiques sont communément pourvoyeurs d´une hospitalisation en psychiatrie [4, 5]. Au Canada, McGuckin *et al*. montrent que 20,1% des patients admis en hospitalisation ont déclaré utiliser du cannabis dans les 30 derniers jours qui ont précédés la première hospitalisation [6]. En France, Zelicourt *et al*. rapportent une proportion d'épisodes maniaques nécessitant une hospitalisation à environ 63,0% [7]. En Tunisie, Dammaka M *et al*. ont identifié cinq variables qui sont significativement associées à la décision d´hospitalisation (symptômes psychotiques, consultation non volontaire, dangerosité pour soi, dangerosité pour autrui, trouble mental majeur) [8]. Au Mali, il n'existe pas d'étude de prévalence des troubles mentaux dans la population générale et l´histoire des soins modernes de santé mentale est relativement récente. C´est vers les années 1960 et 1970 que les tous premiers neuropsychiatres et infirmiers en psychiatrie ont été installés [9, 10]. Le pays compte 0,05 psychiatre pour 100.000 habitants, et seulement 130 lits d´hospitalisation pour 19 000 000 habitants. Selon l´annuaire statistique du système national d´information sanitaire du Mali, le pays a enregistré 2 845 cas de troubles mentaux dans les hôpitaux en 2018. Parmi cette population de patients qui demande des soins en psychiatrie, une franche a nécessité une hospitalisation et dont le profil mérite d´être connu en vue d´organiser les offres de soins sur le territoire. L´objectif de ce travail a été de décrire les aspects cliniques des personnes hospitalisées dans le service de psychiatrie pour trouble psychiatrique de 2014 à 2018.

## Méthodes

**Type d´étude:** Il s'agissait d'une étude transversale réalisée dans le service de psychiatrie du Centre Hospitalier Universitaire (CHU) Point G sur les dossiers de 1105 patients hospitalisés entre janvier 2014 et décembre 2018.

**Cadre de l´étude:** cette étude a été conduite dans le département de psychiatrie du Centre Hospitalier Universitaire du Point G au Mali. Cet hôpital public est situé au centre de la ville de Bamako la capitale du Mali. Le département de psychiatrie à une capacité de 130 lits avec un personnel composé de huit médecins spécialistes, un psychologue clinicien, douze infirmiers spécialisés en santé mentale, trois assistants sociaux. Trois jours par semaine sont consacrés aux consultations ambulatoires. Un service de garde est disponible tous les jours de la semaine pour l´accueil et la prise en charge des urgences psychiatriques.

**Participants:** tous les patients ayant consulté dans le département de psychiatrie au cours de la période de janvier 2014 à décembre 2018 ont été inclus dans notre étude. Les patients dont les dossiers étaient incomplets et/ou les doublons de réhospitalisations au nombre de 44 n´ont pas été comptabilisés dans l´échantillon présenté.

**Taille de l´échantillon:** au cours de cette étude nous avons inclus un total de 1105 dossiers patients ayant consulté au sein du département de psychiatrie pendant la période concernée.

**Sources des données et mesures:** les données ont été recueillies sur la fiche d´exploitation préétablie (questionnaire de collecte de données). Le questionnaire était structuré pour tenir compte des données sociodémographique des patients, la notion de consanguinité entre les parents, l´usage des drogues, les caractéristiques cliniques et le choix du premier recours aux soins.

### Variables

**Les caractéristiques sociodémographiques :**le genre (homme, femme), le statut matrimonial (célibataire, marié, divorcé/séparé, veuf/veuve), le niveau d´éducation (non scolarisé, primaire secondaire, supérieur, école coranique), l´activité professionnelle (agriculteur/éleveur, commerçant, travailleur du secteur informel, fonctionnaire, salarié du secteur privé, femme au foyer/cuisinier, élève/étudiant, sans emploi/chômeur, retraité). L´antécédent psychiatrique (oui, non). La residence (urbaine, rurale). La notion de consanguinité (oui, non, non précisée). L´usage de drogues: tabac, cannabis, alcool, héroïne, cocaïne, tramadol (oui, non). L´origine de la demande de soins (patient lui-même, famille, établissement scolaire, forces de l´ordre, médecin soignant, collègues). Le motif de consultation/hospitalization (agitation, agressivité, consommation de drogues, fugues/errances, homicide, inhibition, insomnie, propos incohérents, rupture du traitement, tentative de suicide, troubles du comportement, autres). Le diagnostic (troubles mentaux organiques, y compris les troubles symptomatiques, troubles mentaux et du comportement liés à l'utilisation de substances psychoactives, schizophrénie, troubles schizotypiques et troubles délirants, troubles de l'humeur (affectifs), troubles névrotiques, troubles liés à des facteurs de stress et troubles somatoformes, troubles de la personnalité et du comportement chez l'adulte). L´outil de certification du diagnostic était la Classification Internationale des Maladies 10^e^ révision (CIM-10)(11). Le type de soins en premier recours (soins traditionnels, soins médicaux modernes, soins confessionnels, non précisé).

**Gestion et analyse des données:** les données recueillies sur le questionnaire ont été saisies et traitées à l´aide du Pack Office Excel 2016 et du logiciel SPSS version 22.0. Le test de khi2 a été utilisé pour la comparaison des données. Les liens entre les valeurs étaient considérés comme statistiquement significatifs au seuil de probabilité de 0,05. Le logiciel Zotero a été utilisé pour la transcription des références.

**Considérations éthiques:** cette étude a été autorisée par l´administration du Centre Hospitalier Universitaire Point G, le chef du département de psychiatrie et la Faculté de Médecine et Odontostomatologie de Bamako. Les données ont été collectées, analysées et utilisées dans la confidentialité. Aucune information pouvant faire le lien entre un patient et ses données n´a été présentée dans ce travail.

## Résultats

**Figure 1:**
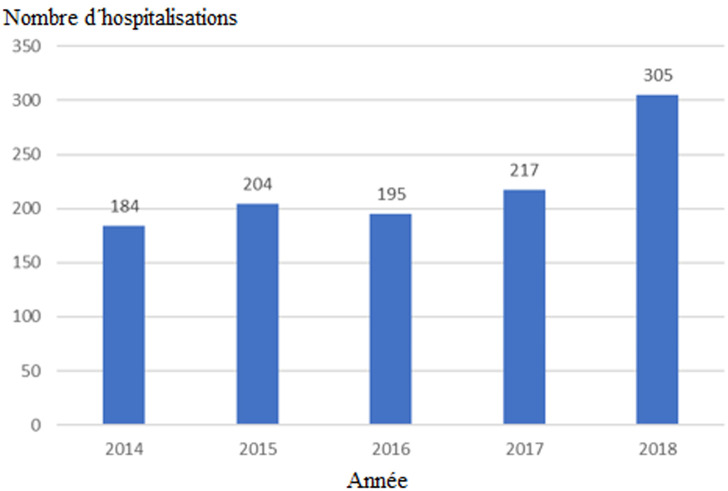
évolution du nombre d’hospitalisations dans le service de psychiatrie du CHU Point G de l’année 2014 à 2018

Sur cinq ans, nous avons recensé 1 149 dossiers d´hospitalisations, dont 1 105 ont été retenu et 44 dossiers ont été rejetés pour insuffisances d´information. La fréquence des hospitalisations était de 10,4% (1 105/10 636) soit 0,01 hospitalisation pour 100 000 habitants. La courbe des hospitalisations était croissante avec un pic observé en 2018 (Figure 1).

**Caractéristiques sociodémographiques de la population d´étude:** l´âge moyen de nos patients étaient de 32,6326± 11,1 ans avec des extrêmes 13 ans et 82 ans. L´âge moyen pour les hommes était de 32,2±10,7 ans et celui des femmes était 35,3±12,8 ans (Tableau 1). Le taux de scolarisation des personnes hospitalisées était de 61,7% (682 /1105). Le niveau primaire était le plus retrouvé parmi les personnes hospitalisées (28,2%) (Tableau 1). La fratrie était précisée pour 655 patients hospitalisés. Les premiers-nés ont représenté 28,1%. La résidence urbaine était retrouvée chez 57,1% des patients hospitalisés (Tableau 1). L´antécédent psychiatrique était retrouvé chez 74,0% des patients hospitalisés (818/105) (Tableau 1). La notion de consanguinité était présentée chez 251 patients hospitalisés soit 22,7% (Tableau 1).

**Tableau 1:** caractéristiques sociodémographiques des personnes hospitalisées dans le service de psychiatrie de 2014 à 2018

N=1105	Total	Homme	Femme	Valeur de p
	N (%)	n (%)	n (%)	
Age moyen (en année)	32,6 ± 11,1	32,2 ± 10,7	35,3 ± 128	
Genre	1105	926(83,8)	179(16,2)	
**Statut matrimonial**				
Célibataire	588(53,2)	553(94,1)	35(5,9)	0,000
Marié	419(37,9)	318(79,9)	101(24,1)	
Divorcé/séparé	72(6,5)	45(62,5)	27(37,5)	
Veuf/veuve	26(2,4)	10(38,5)	16(61,5)	
Niveau d´éducation				
Non scolarisé	316(28,6)	238(75,3)	78(24,7)	0,000
Primaire	310(28,2)	270(87,1)	40(12,9)	
Secondaire	196(17,7)	167(85,2)	29(14,8)	
Supérieur	176(15,9)	158(89,8)	18(10,2)	
Ecole coranique	107(9,7)	93(86,9)	14(13,1)	
**Activité professionnelle**				
Agriculteur/éleveur	171(15,5)	168(98,2)	3(1,8)	0,000
Commerçant	152(13,8)	136(89,5)	16(10,5)	
Travailleur du secteur informel	165(15,0)	154(93,3)	11(6,7)	
Fonctionnaire	57(5,2)	47(82,5)	10(17,5)	
Salarié du secteur privé	123(11,1)	116(94,3)	7(5,7)	
Femme au foyer/Cuisinier	103(9,3)	4(3,9)	99(96,1)	
Elève/étudiant	120(10,9)	110(91,7)	10(8,3)	
Sans emploi/chômeur	208(18,8)	186(89,4)	22(10,6)	
Retraité	6(0,5)	5(83,3)	1(16,7)	
**Antécédent psychiatrique**				
Oui	818(74,0)	694(84,8)	124(15,2)	
Non	287(26,0)	232(80,8)	55(19,2)	0,069
**Résidence**				
Urbaine	631(57,1)	545(86,4)	86(13,6)	0,007
Rurale	474(42,9)	381(80,4)	93(19,6)	
**Notion de Consanguinité**				
Oui	251(22,7)	211(84,1)	40(15,9)	0,726
Non	583(52,8)	492(82,1)	91(15,6)	
Non précisée	271(24,5)	223(82,3)	48(17,7)	

**Usages des drogues parmi les personnes hospitalisées:** la notion usage d´une drogue comme le tabac, le cannabis, l´alcool, la cocaine, l´héroïne au tramadol était retrouvé chez 466 patients hospitalisés soit 42,9% et le genre homme 98,3% de cette population (Tableau 2). La fréquence d´usage du tabac était de 32,6%. L´usage du cannabis a représenté 26,0% tandis que, suivi de l´alcool (15,6%).

**Tableau 2:** tendance de l’usage des drogues parmi les personnes hospitalisées dans le service de psychiatrie de 2014 à 2018 et en fonction du genre

N=1105	TOTAL	Homme	Femme	
	N(%)	n(%)	n(%)	Valeur de p
**Usage de drogues**				
Oui	474(42,9)	466(98,3)	8(1,7)	0,000
Non	631(57,1)	460(72,9)	171(27,1)	
**Tabac**				
Oui	361(32,6)	356(98,6)	5(1,4)	0,000
Non	744(67,4)	570(76,6)	174(23,4)	
**Cannabis**				
Oui	287(26,0)	283(98,6)	4(1,4)	
Non	818(74,0)	643(78,6)	175(21,4)	
**Alcool**				
Oui	172(15,6)	169(98,3)	3(1,7)	0,000
Non	933(84,4)	757(81,1)	176(18,9)	
**Cocaïne**				
Oui	33(3,0)	31(93,9)	2(6,1)	0,209
Non	1072(97,0)	895(83,5)	177(16,5)	
**Héroïne**				
Oui	30(2,7)	28(93,3)	2(6,7)	
Non	1075(97,3)	898(83,5)	177(16,5)	
**Tramadol**				
Oui	51(4,6)	50(98,0)	1(2,0)	0,002
Non	1054(95,4)	876(83,1)	178(16,9)	

**Demandes de soins et motifs de consultation/hospitalisations parmi les participants:** la famille était à l´origine de la demande de soins de 87,5% des personnes hospitalisées dans le service de psychiatrie. L´agressivité le motif de consultation pour 492 des 1105 patients hospitalisés soit 44,5% (Tableau 3).

**Tableau 3:** description des demandes de soins et des motifs de consultation ou des hospitalisations pour les personnes hospitalisées dans le service de psychiatrie

N=1105	Total	Homme	Femme	Valeur de p
	N(%)	n(%)	n(%)	
**Origine de la demande de soins**				
Patient lui-même	8(0,7)	7(87,5)	1(12,5)	0,013
Famille	967(87,5)	815(84,3%)	152(15,7)	
Établissement scolaire	1(0,1)	1(100,0)	0(0,0)	
Forces de l´ordre	26(2,3)	26(100,0)	0(0,0)	
Médecin soignant	101(9,1)	75(74,3)	26(25,7)	
Collègues	2(0,2)	2(100,0)	0(0,0)	
**Motifs de consultation/hospitalisation**				
Agitation	267(24,2)	201(75,3)	66(24,7)	0,001
Agressivité	492(44,5)	435(88,4)	57(11,6)	
Consommation de drogues	35(3,2)	32(91,4)	3(8,6)	
Fugues/errances	40(3,6)	34(85,0)	6(15,0)	
Homicide	5(0,5)	5(100,0)	0(0,0)	
Inhibition	24(2,2)	23(95,8)	1(4,2)	
Insomnie	20(1,8)	15(75,0)	5(25,0)	
Propos incohérents	67(6,1)	59(88,1)	8(11,9)	
Rupture du traitement	21(1,9)	18(85,7)	3(14,3)	
Tentative de suicide	5(0,5)	4(80,0)	1(20,0)	
Troubles du comportement	124(11,2)	96(77,4)	28(22,6)	
Autres	5(0,5)	4(80,0)	1(20,0)	

**Catégories diagnostique parmi les participants:** le groupement diagnostic de schizophrénie, troubles schizotypiques et troubles délirants était retrouvé chez 67,8% des personnes hospitalisées en psychiatrie. Les hommes représentaient 85, 7% et les femmes 14,3% de cette population (Tableau 4). Type de soins en premier recours chez les **participants:** les soins traditionnels étaient le type de soins le plus retrouvés en premier recours parmi les chez les participants (**Tableau 5**).

**Tableau 4:** répartition des personnes hospitalisées par catégories diagnostique

N=1105	TOTAL	Homme	Femme
	N(%)	n(%)	n(%)
**Diagnostics**			
Troubles mentaux organiques, y compris les troubles symptomatiques	82(7,4)	67(81,7)	15(18,3)
Troubles mentaux et du comportement liés à l'utilisation de substances psychoactives	62(5,6)	58(93,5)	4(6,5)
Schizophrénie, troubles schizotypiques et troubles délirants	749(67,8)	642(85,7)	107(14,3)
Troubles de l´humeur (affectifs)	201(18,2)	149(74,1)	52(25,9)
Troubles névrotiques, troubles liés à des facteurs de stress et troubles somatoformes	8(0,7)	7(87,5)	1(12,5)
Troubles de la personnalité et du comportement chez l´adulte	3(0,3)	3(100,0)	0(0,0)

**Tableau 5:** répartition de la population selon le type de soins en premier recours

N=1105	Total	Homme	Femme	P
		n(%)	n(%)	
**Type de soins en premier recours**	**N(%)**			
Soins traditionnels	649(58,7)	535(82,4)	114(17,6)	0,570
Soins médicaux modernes	282(25,5)	241(85,5)	41(14,5)	
Soins confessionnels	10(0,9)	9(90,0)	1(10,0)	
Non précisé	164(14,8)	141(86,0)	23(14,0)	

## Discussion

Cette étude s´était intéressée aux aspects cliniques des personnes hospitalisées dans le service de psychiatrique du centre hospitalier universitaire Point G au Mali. L´étude a montré que les personnes hospitalisées étaient majoritairement des hommes (83,8%) [11] (Tableau 1). Au Maroc, Belghazi *et al*. ont trouvé 77,7% d´hommes [12], au Burkina Faso, Ouedraogo *et al*. ont trouvé 60,0% d´hommes [13], En France, Le Fur *et al*. ont rapporté 63,8% d´hommes dans leur échantillon [14]. Une tendance contraire est rapportée par une étude canadienne où les femmes ont été plus demandeuses de soins auprès des professionnels de santé mentale [15]. Bien que nous ne disposions pas de prévalence des troubles mentaux en population générale au Mali, la plupart des travaux réalisés en milieu hospitalier rapporte cette prédominance des hommes dans le service de psychiatrie [16-18] et pourrait aussi refléter la réalité en communauté. Au Maroc, comme au Burkina Faso, des études en population générale rapportent, à l´inverse des études en milieux hospitaliers, une prédominance des femmes pour des troubles psychiatriques [19, 20]. Aussi, nous pouvons soutenir l´explication que la prédominance des hommes en milieu hospitalier est la traduction du faible accès des femmes aux soins de santé mentale dans le système conventionnel au Mali. La fréquence des hospitalisations était d´environ 10% dans notre étude. Au Burkina Faso, Ouedraogo *et al*. ont trouvé une fréquence de 19,4% [13]. En absence de données sur la santé mentale en population générale au Mali, ce faible taux des hospitalisations en psychiatrie (0,01 pour 100000 habitants) pourrait s´expliquer soit par l´inaccessibilité du système de soins aux personnes malades.

Nous avons constaté une augmentation relative du nombre des hospitalisations au fil des années à l´exception de l´année 2016 (Figure 1). Cette période est aussi concomitante à la dégradation de la situation politique et sécuritaire du pays [21]. L´âge moyen de nos patients était de 32,6 ± 11,1 ans. Les hommes étaient relativement jeunes avec un âge moyen de 32,2 ± 10,7 ans comparé aux femmes chez qui l´âge moyen était de 35,3 ± 12,8 ans. Au Burkina, Ouédraogo *et al*. ont trouvé un âge moyen de 31,06 ± 13,07 ans avec une élévation significative de l´âge chez les femmes (32,01 ± 14,04 ans) comparées aux hommes (30,42 ± 12,33 ans) [13]. Notre population de patients hospitalisés était pour la majorité des célibataires avec plus de 50% (Tableau 1). Une tendance supérieure à celle de notre étude est rapportée par Belghazi *et al*. au Maroc avec une fréquence des célibataires de l´ordre de 80,0 % [12]. Parmi célibataires, les hommes ont représenté plus de 90% contre environ 6% pour les femmes avec une différence statistiquement significative (p=0,000) (Tableau 1). Belghazi *et al*. ont trouvé que 80,3% des hommes hospitalisés étaient des célibataires contre 54,3% de femmes célibataires [12].

Le taux de scolarisation représentait plus de 60% parmi nos patients. Ce résultat est comparable au taux net de scolarisation de l´année scolaire 2009-2010 au Mali qui était de 60,6% [22]. Le niveau d´instruction de nos patients était relativement bas avec une prédominance des patients de niveau primaire (plus de 28%), le niveau supérieur a représenté approximativement 16%. Ce résultat pourrait s´expliquer par des difficultés d´achèvement des études à cause de la maladie mentale [23]. La grande majorité des patients avaient un antécédent psychiatrique avec plus de 70% (Tableau 1). Koné *et al*. ont rapporté la présence des antécédents psychiatriques comme facteur de réadmission à l´hôpital psychiatrique [24]. La vie en milieu urbain est signalée comme un facteur de détresse psychologique [24]. Nos patients hospitalisés résidaient pour la plupart en zone urbaine (plus de 57%) (Tableau 1). Nos résultats pourraient aussi être influencés par le site de recrutement qui était une zone urbaine et plus accessible à cette population.

La notion de consanguinité était trouvée chez plus de 22% des personnes hospitalisées (Tableau 1). Plusieurs études ont invoqué le rôle de la consanguinité dans l´apparition des troubles psychiatriques [25, 26]. Cela pourrait ouvrir comme perspective la mise en œuvre d´étude biogénétiques sur la maladie mentale. L´usage des drogues est fréquent dans la population des personnes souffrantes de troubles mentaux [27-29]. Nos résultats ont montré une forte prévalence de la consommation des drogues par des patients hospitalisés avec plus de 40% (Tableau 2). Les hommes étaient les plus concernés par cette consommation avec plus de 98%. Le tabac était le produit le plus consommé suivi du cannabis et de l´alcool. Ces trois produits sont reconnus pour leurs effets anxiolytiques [30, 31]. Les patients utilisent très souvent ces drogues à but autothérapeutique, pour contenir leurs angoisses. La quête de cet effet pourrait expliquer la tendance des patients à l´usage de ces drogues. Les manifestations des troubles psychiatriques qui aboutissent à une hospitalisation posent souvent un problème de contenance à l´environnement du patient et le patient se montre très souvent opposant aux traitements. La demande de soins est ainsi rarement formulée par le patient lui-même. Nos résultats ont montré que la demande de soins provenait des familles dans la majorité des cas (Tableau 3). En effet, pour beaucoup de patients, il s´agit des états graves où le patient n´a pas la conscience de l´état morbide et donc ne saurait demander les soins. Au Maroc, Belghazi *et al*. affirme que l´hospitalisation psychiatrique concerne les cas graves de schizophrénie, de dépression, et troubles bipolaires [12]. Dans le contexte africain, les demandes de soins psychiatriques sont le plus souvent formulées devant des manifestations socialement peu tolérées, comme l´agitation, l´agressivité [13, 20, 32]. Nos résultats confirment ce constat; nous avons trouvé l´agressivité comme premier motif d´hospitalisation en psychiatrie suivi de l´agitation (Tableau 3).

Nos résultats ont montré que l´entité diagnostic schizophrénie, troubles schizothymiques et troubles délirants était la plus retrouvée parmi les patients hospitalisés en psychiatrie (Tableau 4). Cette entité se manifeste par des symptômes comme le délire, l´agitation, l´agressivité, le déficit cognitif, l´inadaptation sociale et professionnelle [33]. Ces symptômes sont difficiles à contrôler par l´entourage, ce qui pourrait expliquer cette forte prévalence en hospitalisation. Pour Belghazi *et al*. les troubles schizophréniques étaient les plus représentés [12]. Ouédraogo *et al*. ont rapporté la fréquence des troubles psychotiques aigus transitoires, les schizophrénies et les états dépressifs [13]. Les patients avaient eu recours en premier lieu aux traitements traditionnels avant leur hospitalisation dans plus de 58% des cas (Tableau 5). Il n´y avait pas de lien significatif entre le type de recours aux soins et le genre. Le recours aux soins traditionnels est soutenu par les causalités surnaturelles qui sont attribuées à la maladie mentale dans le contexte Africain y compris celui du Mali [10, 34-36]. Cela est une opportunité culturelle pour le Mali de mettre en place un cadre de collaboration entre médecin psychiatres, psychologues et biologistes et pharmaciens pour comprendre les approches sociopsychologiques de la prise en charge, mais également un cadre idéal pour la découverte de principe actif des plantes médicinales ayant une utilité en santé mentale.

## Conclusion

La majorité des personnes hospitalisées était des hommes, jeunes, ayant des antécédents psychiatriques et chez lesquels les diagnostics de schizophrénie, de troubles schizotypiques et de troubles délirants étaient les plus évoqués. Il ressort de cette étude que le premier recours aux soins devant des manifestations psychiatriques reste les soins traditionnels.

### Etat des connaissances sur le sujet

- La stigmatisation et déficit de soins;
- Insuffisance d´étude de prévalence des troubles mentaux au Mali;
- L´histoire des soins modernes de santé mentale est relativement récente; les données sur la consommation des drogues et les troubles mentaux.

### Contribution de notre étude à la connaissance

- Une tendance de la fréquence des troubles mentaux sur cinq ans ;
- La consanguinité en lien avec les troubles mentaux ;
- Mise en exergue des états psychotiques comme dominant l´épidémiologie des troubles mentaux sur cinq ans.
